# Improving Equity Across Cancer Care Continuum in Asia Pacific

**DOI:** 10.1200/GO.23.00056

**Published:** 2023-06-26

**Authors:** Diah Martina, Eva Segelov

**Affiliations:** ^1^ Division of Psychosomatic and Palliative Medicine, Department of Internal Medicine, Faculty of Medicine, Universitas Indonesia, Jakarta, Indonesia; ^2^ Cipto Mangunkusumo National General Hospital, Jakarta, Indonesia; ^3^ Faculty of Medicine, Nursing and Health Sciences, Monash University, Melbourne, Victoria, Australia; ^4^ Faculty of Medicine, University of Bern, Bern, Switzerland

## INTRODUCTION

“The reality today is that who you are and where you live could mean the difference between life and death. It isn't fair. But we can change this (Jeff Dunn, UICC President, 2022).”

**FIG 1:**
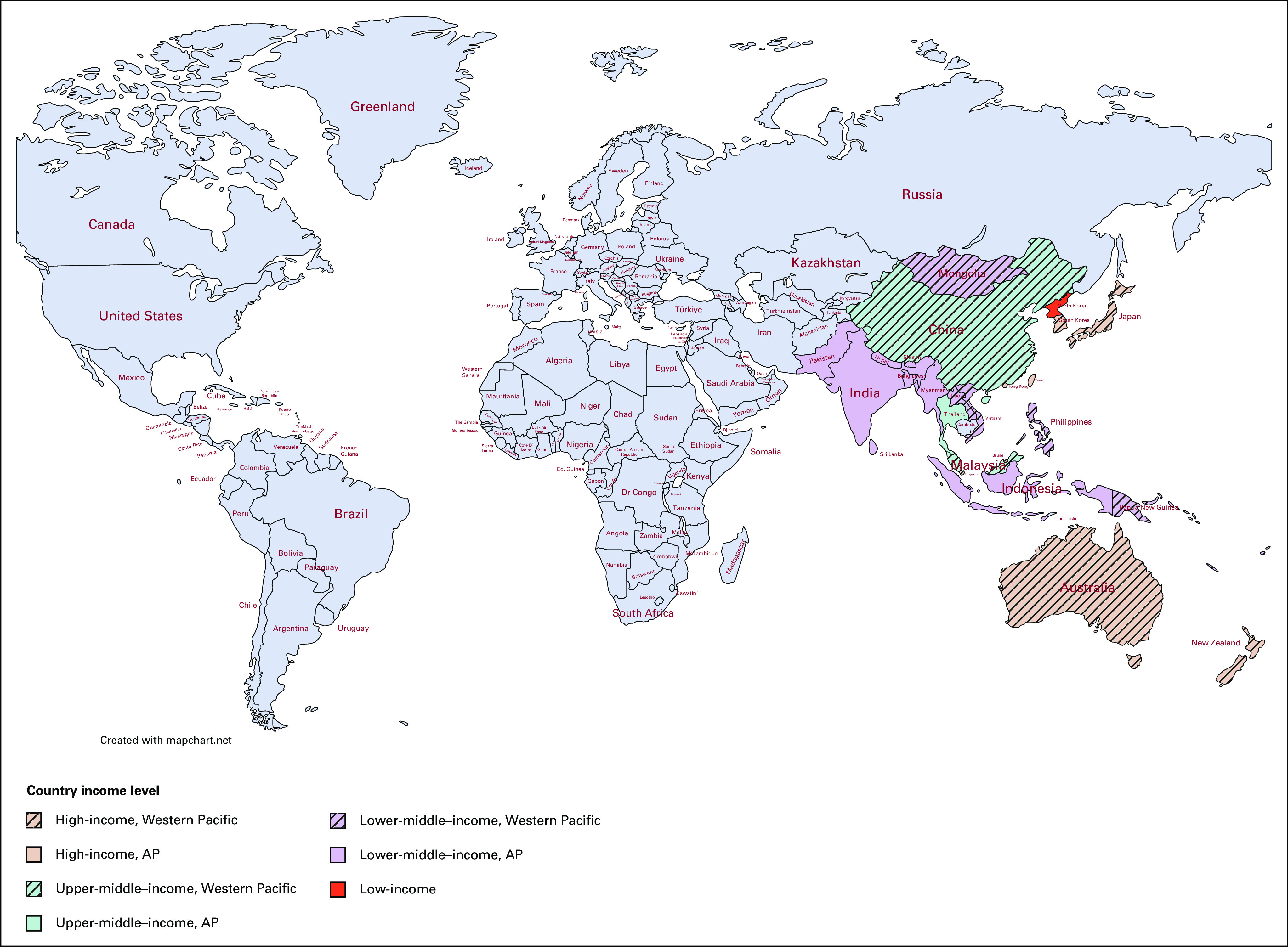
AP countries on the basis of income level.^2,3^ AP, Asia Pacific.

One half of all cancer cases and 58% of cancer deaths occur in Asia Pacific (AP), where more than 60% of the world's population reside.^1^ Moreover, in this region, cancer was the cause of a quarter of deaths because of noncommunicable diseases in 2019, making AP the region that bears the heaviest burden of cancer worldwide.^2^ This is occurring at a time that AP is rapidly evolving a vital role in global health. It is the fastest growing economic region that comprises a diverse group of income countries (Fig 1).^1,3^ The huge divide between rich and limited-resource countries is evidenced by the fact that despite a higher incidence of cancer in high-income countries, survival rates continue to improve. On the contrary, although cancer incidence is lower in low- and middle-income countries (LMICs), mortality is significantly higher.^4^ Given the diverse health systems, particularly in relation to government schemes for financial coverage, the stark reality is that a cancer diagnosis can potentially push families into poverty.^5^

Each of the AP countries and territories has a distinctive cultural, linguistic, and geographic landscape which is reflected in variations in cancer risk factors, incidence, unique needs, and challenges in delivering equitable, high-quality cancer care. To highlight these challenges and increase awareness of the complex landscape of oncology across AP, this special series compiles articles focused on three domains: (1) cancer screening and treatment patterns, (2) culturally sensitive cancer care, and (3) global and regional partnership in cancer care.

## CANCER SCREENING AND TREATMENT PATTERNS IN AP

Primary prevention and early detection are keys to reducing the cancer burden. Therefore, raising public awareness and access to cancer screening and diagnosis needs to be prioritized on the national agenda.^2^ Among the cancers of which early diagnosis has been proven to effectively reduce mortality are lung and cervical cancer, both highly prevalent in the AP region.

Lung cancer is responsible for the most cancer-related deaths worldwide; the Western Pacific Region (Fig 1) contributes to almost half of the global lung cancer cases.^2^ Although smoking cessation is the most effective primary prevention, screening with low-dose computed tomography (LDCT) has significantly reduced mortality in large randomized controlled trials. Nevertheless, only 22% of the countries in the Western Pacific Region have implemented lung cancer screening. Nightingale et al^6^ summarized the numerous barriers and challenges for lung cancer screening in this area, including limited evidence about the effective recruitment strategies of high-risk individuals in the region, resource limitation, and infrastructure challenges. The review addresses the importance of primary care practitioners in encouraging the participation of high-risk individuals, the need to improve health care professionals' competency in interpreting LDCT, and subsequently managing suspicious nodules or incidental findings. Finally, greater advocacy for LDCT within LMICs requires careful consideration of competing health priorities, health system capacity, and local cost-effectiveness analysis of individual countries.

Cervical cancer is a highly preventable cancer but remains one of the most commonly diagnosed cancers worldwide; more than half of all cases occur in AP.^7^ Cervical cancer has been referred to as a disease of inequity, given that up to 90% of mortality occurs in LMICs, where screening remains low.^8^ For example, screening coverage is 4% in the Western Pacific and 18% in Southeast Asia, far below the WHO recommendation of 70% screening coverage of eligible women. Self-collection screening of cervical cancer has been proven effective in high-income countries and has the potential to overcome many of the identified barriers to accessing screening in LMICs, namely eliminating the need for a pelvic examination, a clinic setting, and a trained clinician. A review by Creagh et al^9^ summarized the feasibility and acceptability of self-collection screening of cervical cancer in low-, middle-, and high-income settings across the AP region. This review concludes that screen-and-treat models with point-of-care testing are feasible to implement within low-resource settings but that further capacity building for self-collection requires more data evaluating the sustainability of individual programs and their health economic impact.

Another prevailing cancer in AP is liver cancer, with Thailand, Vietnam, China, and South Korea ranked among the top 10 countries with the highest incidence worldwide. Not only incidence but mortality is also significantly higher in LMIC AP countries, where the modern standard-of-care therapeutics, such as combination of atezolizumab and bevacizumab, are not routinely accessible.^10^ Charonpongsuntorn et al^11^ conducted the first multicenter prospective study to generate local data regarding the efficacy and safety profiles of this combination therapy among Thai patients with unresectable hepatocellular carcinoma. This study showed that combination therapy could improve patients' survival and quality of life and confirms the efficacy of this therapy beyond high-income countries. Further advocacy for adoption in Thailand and other limited-resource settings should now focus on the health economic impact.

## CULTURALLY SENSITIVE CANCER CARE IN AP

To enable equitable access to cancer care, every patient should be offered the opportunity to be involved in the decision making around their care. Meaningful engagement requires patients to have a sufficient understanding of their illness; however, providing medical information and sharing decision making are culturally sensitive processes for both the affected individual and their families and carers. Asian countries are primarily collectivist-oriented, where care for an individual is viewed mainly as a family task.^12^ Therefore, medical decision making is often family-centered, concealment of information is common, and social harmony is often valued over individual autonomy. Additionally, if a paternalistic communication style prevails among doctors, patients may have little involvement in decision making.^13^ Mori et al^14^ reviewed evidence on the communication practices with patients with cancer and their families in the AP, showing that despite the traditional scenarios of implicit communication, partial or nondisclosure of information, and family-centered decision making, recent studies show a gradual shift toward open communication in cancer diagnosis, prognosis, advance care planning, and end-of-life discussions. This review has implications for the cancer care of AP populations both within and outside the region. Such cultural sensitivity requires health care professionals to understand the common features of the patients' culture while avoiding stereotypical characterizations. The development of communication tools with strategies to acknowledge these cultural considerations has been shown to be helpful.

Understanding the local culture not only facilitates communication with individual patients but can build partnerships with communities, optimizing their role in supporting cancer care in the public setting. Eng et al^15^ shared the experience of recognizing Indonesia's unique social system and local culture to facilitate a community approach to palliative care. Indonesia is the fourth most populated nation worldwide, with more than 16,000 islands. More than 70% of Indonesian patients with cancer are admitted in their advanced stage, and only 1% can access palliative care.^16^ Because of the limited and unevenly distributed palliative care in health care facilities, most palliative cases in the community rely on charity and activities from various nongovernmental organizations.^17^ Eng's report focuses on the unique Indonesian philosophy of gotong royong or mutual assistance, which signifies social solidarity within the smaller bubbles of the community, which was identified as an enabler of a compassionate community in Jakarta.

One of the most important elements of culture is language. Equal access to care for every patient with cancer requires careful consideration and targeted efforts for linguistically diverse populations. When the language of care provision is not the same as the patient's primary language, it has been shown that patients tend to be more vulnerable to experiencing inequities.^18^ A survey among Tamil and Sinhalese patients with breast cancer in Sri Lanka, a country with multiethnic (Sinhalese, Tamils, Moors, and Burghers) and multilinguistic societies with three different official languages (Sinhalese, Tamil, and English), is reported in this Special Edition. Thanabalasingam et al^19^ showed that patients who speak the same language as their health care providers showed a higher level of satisfaction with the care received.

Alongside the need for the provision of language-concordant care is the provision of high-quality, freely accessible and culturally congruent health information. This is especially true among indigenous populations who are at higher risk of significant disparities in cancer care around the world.^20^ Diaz et al^21^ audited Australian online information resources to explore the publicly accessible information about cardiovascular health after cancer, an issue that is important for survivorship care, particularly for indigenous Aboriginal and Torres Strait Islander people. This study showed that among the dearth of Australian online information resources on cardio-oncology, none was directed at, designed, or even included Aboriginal and Torres Strait Islander people. Given the persistent inequalities in cancer and cardiovascular disease outcomes for Aboriginal and Torres Strait Islander people, the development of resources that take into consideration their readability, understandability, actionability, and inclusivity among Aboriginal and Torres Strait Islander people is imperative.

## GLOBAL AND REGIONAL PARTNERSHIP IN CANCER CARE IN AP

Over the past decades, the pace of innovation in cancer care has been remarkable, spanning novel diagnostics to broad therapeutics. Despite these significant advances, disparities remain wide, with huge variations of needs and health system capability across the AP. Koczwara et al^22^ reported gross inequities in cancer survivorship care and research across the Indo-Pacific. Concern for progression, recurrence, access to care, and coordination of care were among the most reported unmet needs in LMICs. Pleasingly, the study also outlines emerging regional partnerships in survivorship care delivery and research development.

To learn more about the diverse needs and landscapes of oncology in the AP, ASCO launched the Asia Pacific Regional Council (APRC) in 2019 to advise on the member needs and encourage the involvement of regional cancer health care professionals in ASCO's global activities. Highly prioritized was the need to create leaders in the region. Understanding that the traditional Western leadership program may not be well suited to an Asian context, a culturally modified training program was deemed essential. de Guzman et al^23^ shared the experience of the collaboration between the APRC and ASCO in adapting the leadership development program through project-based learning. The paper outlines initiatives relevant to other regions when creating a culturally adapted leadership pathway that fits best with local context and culture.

The collaborative effort between ASCO and AP countries through APRC went beyond the adaptation of a training program to improving the quality of cancer care in medical institutions in LMICs. Wong et al provided a report of the international cancer corps program in 2019, where ASCO collaborated with the team in Sarawak Malaysia to conduct palliative care educational programs, which resulted in positive changes in the practice of oncology care in Sarawak General Hospital.^24^ Trainer skills were taught in the trainer program, and a reservoir of local health care champions was built to ensure the sustainability of the palliative care service in Sarawak. The collaborative effort also led to translational work of the education materials, ensuring language is not a barrier in learning palliative care. Yip shared a similar collaborative effort between professionals from high-income countries working with colleagues from the Solomon islands, a resource-limited country in South Pacific aimed toward establishing a sustainable oncology unit.^25^ This collaboration provides a blueprint for other startup units in the region.

Finally, evidence-based, high-quality cancer care depends on high-quality research. Currently, cancer research is heavily skewed toward high-income countries, with little research conducted in, and relevant to, the problems of LMICs. Day et al^26^ shared the complexities of coordinating multinational clinical trials in AP, because of cultural, linguistic, and economic diversity. Among the challenges were regulatory complexity, communication and logistical barriers, limited funding and resources, disparate experience with trial conduct and trial infrastructure, recruitment holds because of changes in local laws (eg, for biospecimens) and patient attrition, and disruptions caused by the COVID-19 pandemic. Despite all, significant opportunities are identified, and this paper highlights the potential contributions of opening up trials to the AP population, not only for participation but also for leadership.

It is evident that closing the cancer care gap, in a region so rich in culture and language, with a clear distinction between high- and low-income countries, requires significant attention and resources, along with support, collaboration, and cultural humility. We hope this issue of *JGO* will cultivate many more collaborative efforts in the region so that high-quality cancer care can be provided to the many of millions of people wherever they live.
